# Tincture Gelseminum

**Published:** 1877-10

**Authors:** T. J. Fentress


### TINCTURE GELSEMINUM.

*Editor Medical Brief :* Having used this agent for over twenty years in an active practice, we are led to state that we have not a more powerful and reliable remedy in the *materia medica*, when given in accordance with indications demanding its use. Take, for example fever or inflammation, with irritation of the nervous system, a strong tendency to determination to the brain, flushed face, bright eyes, contracted pupils, hot skin, and gelseminum is the remedy *par excellence.* It is positively contra-indicated where the countenance is blanched or expressionless, eyes dull, pupils dilated, skin cool, or imperfect capillary circulation, in which conditions even small doses are apt to prove fatal.

Children, from their immature nervous system, are especially liable to convulsions while suffering with fevers of a high grade, or any sources of irritation. Hundreds of such cases have fallen to our lot; and where the indications were heeded, this remedy brought the answer every time. When the urinary secretions are scanty and high-colored, leaving a burning, smarting, or pain, after micturition, gelseminum, with spts. nit. dulc., gives speedy relief, and is really a blessing in disguise to the suffering patients. In dysuria from stricture, its action is prompt and reliable.

We prefer a saturated green root tincture, made with alcohol of about 76 per cent. T. J. FENTRESS, M. D.

